# 1786. Characterizing Antibiotic Use in Patients with Hematologic and Oncologic Malignancies Transitioned to Comfort Measures Only (CMO) During Hospital Admission

**DOI:** 10.1093/ofid/ofac492.1416

**Published:** 2022-12-15

**Authors:** Michelle Chan, Dana Pepe, Preeti Mehrotra

**Affiliations:** Beth Israel Deaconess Medical Center, Boston, Massachusetts; Beth Israel Deaconess Medical Center/Harvard Medical School, Boston, Massachusetts; Beth Israel Deaconess Medical Center/Harvard Medical School, Boston, Massachusetts

## Abstract

**Background:**

Antimicrobial treatment at the end of life is common. However, there is limited evidence on whether antimicrobial use during hospice care provides symptom palliation or contributes to discomfort. We sought to further investigate antimicrobial use at the transition to CMO in immunocompromised patients with active malignancies.

**Methods:**

We conducted a retrospective cohort study of patients with active malignancies over 18 years of age admitted to Beth Israel Deaconess Medical Center from 3/1/2018 to 3/1/2021, who were transitioned to CMO. From hospital databases and from subsequent chart review, we examined: receipt of antimicrobials at any point in the admission; indication for antimicrobials; timing of CMO order; underlying hematologic/oncologic diagnosis; and concomitant COVID-19 diagnosis. Among the patients who received antimicrobials, we identified patients on antibiotics 48 hours prior to and after CMO order placement. Patients were excluded if CMO orders were reversed.

**Results:**

384 patients met study criteria. The mean age was 67 years, and 50.5% patients were female. 31.8% of patients carried a hematologic malignancy diagnosis while the remaining had solid tumor diagnoses. 88% patients received antimicrobials at any point during their hospitalization. Of the patients who received antimicrobials during their admission, 84% received them in the 48 hours prior to transition to CMO and 15.3% continued receiving antimicrobials after CMO transition. Patients received a mean of 5.7 antibiotics. Most common indications for antimicrobials included pneumonia (22.2%), intra-abdominal infections (13.6%), sepsis (13.3%), and prophylaxis (3.5%), but most (39.8%) received antimicrobials for more than one infection or indication.

**Conclusion:**

Hospitalized patients with active malignancy at the end of life are heavily exposed to antimicrobials. The majority of patients transitioned to CMO receive antimicrobials during their last hospital admission, and a significant subset (15.3%) continue antimicrobials after placement on CMO. Further studies are needed to investigate the potential benefits and harms of continuing antimicrobials in CMO patients, as decisions are complex and individualized.

**Disclosures:**

**All Authors**: No reported disclosures.

